# The Prolongation of Useless Lives

**Published:** 1913-03-08

**Authors:** R. Chambers Norman

**Affiliations:** Secretary-Superintendent. Royal Alfred Hospital, Melbourne.


### The Prolongation of Useless Lives.

*To the Editor of* The Hospital.

Sir,—Although merely a layman, I have been so long associated with hospitals that I can claim to express an opinion on certain problems in connection with the treatment of patients therein that have been frequently brought under my notice, causing me much perplexed thought, and evoking frequent discussions with my professional co-workers. I allude to those numerous cases where patients (mostly the victims of heart complaints) become moribund, but are brought back to life by the prompt and efficacious treatment they receive from the hospital staff, which may take the form of injections of strychnine, inhalation of oxygen, and the free administration of stimulants. Now, the question has often occurred to me, Is the medical man always justified in striving to prolong life to its utmost limit by recourse to heroic measures, or would it not very often be both more merciful and expedient to let the sufferer pass quietly out of life when the organic condition is utterly hopeless? I have used the words, "useless lives," to indicate that class who through physical enfeeblement have become a burden to themselves and others; who desire death, and look forward to it as a happy release; whose recurrent attacks of illness bring them back time after time to our hospitals, till they either come in for the last time to pass out through the mortuary, or die suddenly, it may be, on the street, in a miserable home, or friendless in lodgings. I have known many such cases, but not one to express gratitude for the artificial prolongation of their miserable lives, while not a few have come very near cursing the zeal and energy of doctors and nurses in multiplying their death agonies. The question arises, Is this sort of thing right either in regard to the patient or the hospital?

My own opinion dictates a double negative. Speaking personally, I should not desire to be resuscitated after such a fashion, were I the unfortunate victim of an incurable and painful disease.

Then our hospital beds are by this means too often occupied by persons who at best can only be relieved instead of by those that might be cured, while by having frequent recourse to oxygen and stimulants for the same patient money is spent unprofitably. Do not let me be misunderstood, for I recognise there may be circumstances under which neither effort nor money should be spared in the prolongation of human life, but where the latter can serve no useful end, and is not desired by patient, relatives, or friends, an excess of zeal in the direction indicated is rather to be deprecated than commended.

There is another phase of this question that also deserves serious consideration, and that is to what extent is the use of expensive drugs, and stimulants like champagne, justifiable in our voluntary hospitals?

The writer can recall a case that occurred in a Melbourne hospital where a typhoid patient was given at the institution's expense champagne to the value of £7 10s.

After hovering between life and death for some weeks this patient recovered, and "fiz," on which he was kept alive for days, got the credit. On the other hand, I have known champagne freely administered in very many cases that have not recovered, though possibly it may have served to prolong the patient's life. But simple arithmetic must convince everyone that if a hospital has a limited income and spends an undue amount of it on costly curative agencies its benefits will be more restricted than one where all-round moderation and economy are observed.

Hence I think public hospitals should act on utilitarian principles, and study "the greatest good for the greatest number."—Yours faithfully,
R. Chambers Norman,
Secretary-Superintendent.

Royal Alfred Hospital, Melbourne.

